# Consumer Preferences and Willingness to Pay for Mud Crabs in Southeast Asian Countries: A Discrete Choice Experiment

**DOI:** 10.3390/foods10112873

**Published:** 2021-11-19

**Authors:** Zubayer Sayeed, Hiroaki Sugino, Yutaro Sakai, Nobuyuki Yagi

**Affiliations:** Graduate School of Agricultural and Life Sciences, The University of Tokyo, Tokyo 113-8657, Japan; priyo19018@gmail.com (Z.S.); a-sugino@g.ecc.u-tokyo.ac.jp (H.S.); a-sakai@g.ecc.u-tokyo.ac.jp (Y.S.)

**Keywords:** consumer preference, mud crab, willingness to pay (WTP), country of origin, Southeast Asia, Singapore market, discrete choice experiment (DCE)

## Abstract

The mud crab *(Scylla serrata)* is an economically important species found in the mangroves and estuaries of tropical countries and is a popular seafood product in the coastal areas of Southeast Asian markets. The aim of this study is to identify factors affecting consumer preference of mud crabs, and to understand consumers’ willingness to pay (WTP) for these factors in a Singapore market where imported mud crabs from other Asian countries are sold. The results showed that the country of origin matters to participants, affecting purchasing decisions, and consumers were willing to pay approximately 16.48 SGD (11.49 USD, the average price of four shops: 35.55 USD/kg for one mud crab) more if the mud crabs were from Sri Lanka if compared with Indonesian or Cambodian mud crabs. Moreover, consumers were willing to pay 25.08 SGD (17.48 USD) more if the mud crabs were alive and 13.40 SGD (9.34 USD) less if the mud crabs were frozen compared with fresh, but not alive, mud crabs. Furthermore, consumers’ preference in mud crab was associated with the consumers’ religion. Some groups of consumers preferred female mud crabs with eggs over male mud crabs, while other groups preferred male crabs. The results identified diverse consumer preference of mud crabs and, therefore, could provide new insights that are useful for sustainable consumption of mud crabs in the region.

## 1. Introduction

Mud crabs *(Scylla serrata)*, also known as mangrove crabs, are found mainly in shallow mangrove areas, nearby subtidal and muddy intertidal environments. They are one of the economically important species found in mangroves and estuaries of tropical countries [1]. Mud crabs are an essential profitable seafood product in coastal areas of Southeast Asian countries [2]. Due to buyers’ demand in recent years, the crab business is increasing [3], and the prices of the mud crabs are getting higher. During the peak season, wholesale prices are usually around 30 USD/kg [4]. USA, China, South Korea, Thailand, and Japan are the top five consumers [5].

Mud crabs are primarily caught in countries such as Thailand, Malaysia, Singapore, the Philippines, and Indonesia [4]. Mud crabs in Indonesia, starting in the early 1980s, are an essential seafood product [6] and today Indonesia is one of the countries with the highest production of mud crabs [7]. A large percentage of Indonesian mud crabs are exported live to Taiwan, Malaysia, and Singapore [8]. For Singapore, mud crabs imported from Sri Lanka are commonly found in restaurants and recognized as Sri Lankan crab. Mud crabs in Cambodia are sold straight to international or domestic markets without the processing of their meat. The Ministry of Agriculture has heavily promoted the crab bank system (a form of community-based fisheries resource management) in Cambodia. According to Cambodia government statistics in 2020, the total aquaculture production of mud crabs was around 375 mt.

Carlucci et al. [9] reviewed 49 articles related to the purchasing behavior of seafood products. The habitual factors they have listed were fish availability, price perception, self-efficacy in the fish preparation process, convenience perception, fish-eating habits, health beliefs, and sensory perception. Other attributes include production method, country of origin, preservation method, product innovation, packaging, and eco-labeling. In recent times, consumers have been concerned about the condition of the seafood product, especially live and frozen products. A study related to consumption of different sexes of crabs was conducted by a data journalist [10] in the USA and it concluded that USA consumers prefer to eat male crabs over female crabs because of the size and demand. Due to this preference, female crabs cost less than male crabs in the USA. Furthermore, it was also found that USA consumers did not like female crabs due to the presence of roe.

The aim of this study is to identify factors affecting consumer preference of mud crabs, to understand the preference of consumers to choose specific sexes of mud crabs, and also to know the willingness to pay (WTP) for mud crabs from Southeast Asian countries sold in Singapore markets. Singapore was chosen to be the research area over other Southeast Asian countries because Singapore is the most popular in terms of importing mud crabs for their famous dishes, such as chili crab and pepper crab, and, according to FAO, GLOBEFISH [4], the consumption rate per capita is 1 kg/per year. Consumption of beef, chicken, pork, and seafood in Singapore in 2020 was 24,976 metric tons, 205,639 metric tons, 123,625 metric tons, and 124,554 metric tons, respectively [11]. Previously, there have been studies related to the WTP for seafood [12,13], but there is a lack of research related to the willingness to pay for mud crabs. Knowing about mud crabs will help to set a pricing strategy for developing countries that are selling or willing to export mud crabs to different countries, especially in Southeast Asia. This paper analyzes the consumer preference and willingness to pay for mud crabs in Singapore markets imported from other countries. Particular focus is placed upon the consumer preference in both male and female mud crabs.

## 2. Materials and Methods

### 2.1. Survey Design

Online surveys were conducted in Singapore between 1 March and 20 April 2021. The authors have contracted a marketing research company named Apeiron, based in Singapore. The survey was designed by the authors and Apeiron distributed the surveys to respondents in Singapore. Survey questions were designed to be similar to past studies related to consumer preference of seafood [12,14]. One closed-ended question is asked at a time, and a photograph of both male and female mud crabs were used to give a general idea about the different sexes of mud crabs to respondents. Stratified sampling was used to identify a group of respondents who consume seafood products on a regular basis. As compensation for filling in the surveys, 5 SGD (3.49 USD) was distributed to each of the respondents to increase their willingness to participate and improve the accuracy of the survey.

Two surveys were conducted in Singapore. The first was a preliminary survey (*n* = 16) and the second was the main survey (*n* = 312) to calculate the WTP. The preliminary survey was conducted to find important attributes and their levels for a discrete choice experiment (DCE) in the main survey. Previous studies suggested that the country of origin was one of the most important attributes in consumer preferences research [15,16,17,18,19]. Another important attribute related to the consumer preference research is the price, which is a negative attitude factor for fish consumption [20]. The preliminary survey was designed to capture proper levels of these attributes.

**Figure 1:**
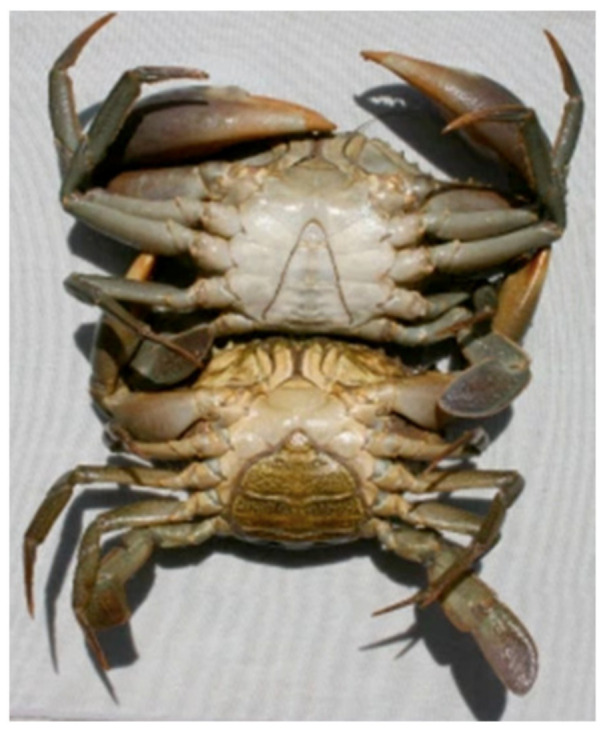
Picture of mud crabs (upper one: male mud crab, lower one: female mud crab) product subject to study.

The preliminary survey consisted of photographs of both male and female mud crabs to give a general idea of both sexes of mud crabs (Figure 1), along with 6 general questions, such as “whether they like to eat mud crabs” in (Q1), “major factors that they consider while choosing mud crabs (Q2)”, those who answered ‘country of origin’ from the selected (Q2) only were asked which countries’ mud crabs do they prefer (from the selected countries given by the authors from the top most producing countries of mud crabs) (Q2.1), “what condition of mud crabs do they prefer while buying them (Q3)”,“if they buy mud crabs in Singapore (Q4)”, and “where do they consume mud crabs in Singapore (Q5)”.

After the attributes were selected using the results of the preliminary survey, the main survey was conducted as a DCE to measure the WTP mud crabs based on selected attributes and the main survey was constructed.

A DCE is a well-recognized way to evaluate utilities for each level or attribute [21]. To identify factors contributing to consumer preferences, an alternative way is to use actual market data. However, acquiring market data is not always easy. DCEs have usually been applied to study seafood preferences [22]. Thus, the DCE method was chosen to conduct this research.

The second survey had three sections that included 28 questions in total.

The first section consisted of 12 general questions, such as “do they eat crabs or not (Q1)”, “the reasons behind saying ‘no’ in Q1 (Q2)”, “the frequency of mud crab consumption (Q3)”, “the places or locations of mud crab consumption (Q4)”, “the locations or places where the consumers buy mud crabs in Singapore (Q5)”, “the purpose of consuming mud crabs (Q6)”, “how the consumers like to consume mud crabs (Q6.1)”, “consumers’ preference of male or female mud crabs (Q7)”, “whether consumers prefer mud crabs from specific countries (Q8)”, “consumers’ preference of eating fish other than mud crabs (Q9)”, “the reason for answering fish with or without eggs (Q9.1)”, and “the alternatives to mud crabs (Q10)”.

The second part consisted of 9 questions related to the DCE, and factorial design was used to generate choice sets by R. The third section consisted of 6 demographic questions. The third section of the survey was on demographic attributes, such as “age (Q1)”, “gender (Q2)”, “religion (Q3)”, “profession (Q4)”, “number of people consumers live with (Q5)”, and “monthly household income (Q6)”. In Singapore, the ethnicity of the respondents is mostly Asian, but food preferences may vary among Asians according to their religions. In this research, therefore, information on the religion of each respondent is collected.

A total of 312 people were surveyed for the main survey.

### 2.2. Attributes and Levels Identification

Four attributes for the DCE were selected to construct choice sets in the main survey. These were country of origin; price; condition; and sex of the mud crabs (see Table 1). Sri Lanka was selected because it is the most popular country of origin of mud crabs among the respondents of the preliminary survey. Indonesia was selected as it was the second largest exporter of processed/frozen crab after China, and a large percentage of the Indonesian mud crabs are exported to Singapore. Cambodia was selected to cover another geographical area. In terms of price, 3 price levels were selected that were consistent with the prices given on the website of the shops. 50 SGD, 60 SGD, and 70 SGD (34.86 USD, 41.83 USD, and 48.80 USD) (USD = 1.4344 SGD (As of 1 April 2021, Xe currency converter)) were picked for the DCE survey. The condition and the sex of the mud crabs were also selected.

Given the number of attributes and levels, the possible number of all combinations of the attributes and levels was 81 (3 × 3 × 3 × 3 = 81). A fractional factorial design was used to generate choice sets by R software to minimize the respondents’ burden. A total of 9 choice sets were constructed. Each choice set consisted of 3 alternatives and an opt-out alternative. In each choice set, respondents were asked to select one of the four alternatives, three choice sets and a ‘Neither’ option (see Table 2).

## 3. Discrete Choice Analysis: Conceptual Framework and Statistical Model

DCE analyses were used to conduct this study, which has already been applied to various studies related to consumer preferences [12,13]. In this study, a conditional logistic regression model was used to conduct the analysis. Logit models are usually used in consumer behavior research fields [14]. Statistical analysis of the DCE and WTP was completed using the apollo packages. The apollo package presented the process of DCEs using R, which enabled the authors to estimate the DCE model and WTP [23].

The discrete choice analysis was conducted utilizing the results from the choice experiments [12,14,24,25]. The discrete choice analysis is an analytical method to convey respondent utility based on random utility theory (RUM). In random utility theory, it anticipates that respondents made their purchasing decision by comparing the utilities obtained from the combination of the attributes of products. Assume the utility *U* can be expressed by the following equation when a respondent *n* selects *j* among *J* alternatives:

(1) $$U_{nj}=V_{nj}+\varepsilon_{nj}=X_{nj}^{\prime }\beta +\varepsilon_{nj},\;j=1,\;\ldots ,\;J$$

where $V_{nj}$ is the observable product attribute of the utility, *ε* is the error term, ***X’*** is a vector indicating the level of the attributes, and *β* is the parameter vector of the attributes. $\varepsilon_{nj}$ is the utility from unobservable component. The fact that respondent *n* chooses option *j* means that the utility will be higher than choosing the other options. Thus, the probability of choosing option *j* can be expressed as follows:

(2) $$Pr\left(j\right)=Pr(U_{nj}>U_{nm})\;\;\;\;\;\;\;\;=Pr\left(V_{nj}+\varepsilon_{nj}>V_{nm}+\varepsilon_{nm}\right)\;\;\;\;\;\;\;\;\;\;\;\;\;\;\;\;=Pr(V_{nj}-V_{nm}>\varepsilon_{nm\;}-\varepsilon_{ij}),\;\forall \;m\;\neq \;j\forall \;m\;\neq \;j$$

The above equation is the probability of choosing option *j* in discrete choice analysis. Hence, if a linear function for the observed component *V* denoted and predicted that the probability distribution i.e., the Gumbel distribution, of utility ε for the unobservable component is similar for all of the other alternatives. This means that the errors in the Gumbel distribution are all identically distributed. So, the probability that the respondent *n* chooses option *j* can be stated by the following conditional logit model Equation (3).

(3) $$Pr\;\left(j\right)=\frac{\exp\left(V_{nj}\right)}{\sum_{J=1}^{J}\exp\left(V_{nj}\right)}=\frac{\exp\left(X_{nj}^{\prime }\beta \right)}{\sum_{J=1}^{J}\exp\left(X_{nj}^{\prime }\beta \right)}$$

It is well-known that the conditional logit model requires the Independence of Irrelevant Alternatives (IIA) condition. This states that the odds of selecting two alternatives is independent from other alternatives. This is a restrictive assumption to make for studies that use observational data because, in reality, there may be unobservable product attributes in the product choices that will make some alternatives closer substitutes than others. If so, the existence or absence of such alternatives will affect the odds of selecting two alternatives. Nevertheless, in this paper, this assumption seems reasonable, since the choices on the choice sets are generated by the algorithm, where there are no unmeasured characteristics.

Dummy coding was used, with the following base categories being: Sri Lanka, fresh, but not alive, mud crabs, and male mud crabs. Therefore, the explanatory variables in the model that was used in the paper were country of origin (Cambodia, Indonesia), sex of the mud crabs (female mud crab with eggs and female mud crab without eggs), and condition of the mud crabs (alive mud crabs and frozen (whole) mud crabs), as well as the standard alternative specific constant (ASC) for choice 1–3 option. The magnitude of the estimation of each parameter was calculated by the marginal willingness to pay (MWTP) which was estimated as the change of price that offsets a change of unit in the attribute, as well as keeping the total utility constant. Denote the utility for an individual respondent *n* from an option *j* is written as:

(4) $$U_{nj}=\alpha +\beta_{1}Cambodia_{j}+\beta_{2}Indonesia_{j}+\beta_{3}Female\;with\;eggs_{j}+\beta_{4}Female\;without\;eggs_{j}+\beta_{5}alive\;mud\;crabs_{j}+\beta_{6}Frozen\;\left(whole\right)mud\;crab_{j}+\beta_{7}Price_{j}+e_{nj}$$

where $U_{nj}$ represents the utility to choose for mud crabs chosen by the participant j; α represents the alternative specific constant (ASC), denoting the likelihood of the consumption of mud crab choices’ option relative to the opt-out (neither) option (specified using dummy coding); $\beta_{1-7}$ represents the source of the country of origin, sex of the mud crabs, conditions of the mud crabs (three coefficients for four categorical levels), and the price attributes, respectively; $e_{nj}$ represents a random variable, whose value is not observed by the researcher.

Fisher’s exact test is a statistical test used to determine if there are any non-random relationships between two categorical variables. A contingency table, or cross-tabulation, is used to summarize the correlation between categorical variables. Subsequently, Fisher’s exact test was conducted to check if there was any relation between religion and the consumption of different sexes of mud crabs.

To look more closely at the details, the post hoc analysis (adjusted residual analysis) is used to see the significance of each cell between religion and the preference of different sexes of mud crabs. The adjusted residuals that are more than 1.96 in the table indicate that the number of cases in that cell is significantly larger than estimated, if the null hypothesis were rejected with a statistically significant *p*-value of less than 0.05. Moreover, an adjusted residual value of less than −1.96 indicates that the number of cases in that cell is significantly smaller than expected, if the null hypothesis were rejected [26].

## 4. Results

### 4.1. Respondents’ Characteristics

The data were collected from 312 people in total. All data were checked first by the marketing research company Apeiron and later by the authors before carrying out the analysis. All of the data from the 312 respondents were included in the analyses.

Table 3 shows the demographic characteristics of the participants (*n* = 312). 60.6% are male, and 39.4% are female participants. 39.7% are between the ages of 26–35, and 26.3% are between 35–45. In terms of religion, both Buddhism and Christianity are 24.7%, followed by Muslims (14.7%), No religion (13.8%), Hinduism (12.2%), Taoism (4.2%), Sikhism (1.3%), Atheism, and Judaism (0.3%). The majority of the participants are employed (62.2%), followed by students (18.9%), self-employed (12.5%), and unemployed (5.8%). 43.9% of the respondents live with family members of 2–3 people, and 41.3% live with 4–5 people. 20.8% of the respondents’ income is less than S$5000.

### 4.2. Preference for Mud Crabs and the Willingness to Pay

The conditional logit model results are shown in Table 4. All coefficients were statistically significant at *p* < 0.01, except for ‘female mud crab with eggs’ and ‘female mud crab without eggs’. The positive symbol of any coefficient in the estimate meant that the respondents preferred that particular attribute level compared to its reference level, and the negative symbol meant vice versa. The respondents preferred mud crabs from Sri Lanka compared to the mud crabs of Indonesia and Cambodia. Furthermore, respondents preferred alive mud crabs compared to frozen or chilled mud crabs. Lastly, no statistically significant results were obtained on the preference of male mud crabs over female mud crabs with or without eggs.

Table 4 also shows the marginal willingness to pay (MWTP) for each attribute, which was analyzed to see how much the participants were willing to pay for the mud crabs based on the level change of the attribute. The participants were willing to pay around 17 SGD (11.85 USD) less for mud crabs from Indonesia and Cambodia than Sri Lankan mud crabs. Furthermore, participants were willing to pay 25.1 SGD (17.5 USD) more for live mud crabs and 13.4 SGD (9.34 USD) less for frozen mud crabs.

The result of the *p*-value in Fisher’s exact test (Table 5) was 0.023, which was statistically significant (*p* < 0.05), meaning the null hypothesis was true. Religion was associated with the preference of consumption of male and female mud crabs. Additionally, after Fisher’s exact test, post hoc testing was conducted to further investigate and determine which religion of respondents prefer which sexes of mud crabs.

A contingency table (Table 5) was constructed between religion and the consumption of male or female mud crabs. Thereafter, Fisher’s exact test was conducted to check if there was any relation between religion or consumption of different sexes of mud crabs. The result of the p-value in Fisher’s exact test was 0.023, which was statistically significant (*p* < 0.05), meaning the null hypothesis was true. Religion was associated with the preference of male and female consumption of mud crabs. Additionally, after Fisher’s exact test, post hoc testing was conducted to further investigate and determine which religion of respondents prefer which sexes of mud crabs.

Table 6 shows the adjusted residual analysis between the religion and preference of consumption of mud crabs. Respondents from the Christian religion prefer to eat female mud crab without eggs. On the other hand, Hindu respondents prefer to eat male mud crabs.

Table 7 shows the preference of the country of origin of mud crabs that participants chose to eat. 308 respondents answered this question, and the majority of the respondents preferred to eat Sri Lankan mud crabs, followed by Singapore, Malaysia, Cambodia, and Indonesia. It was found from the main survey that the main reason for selecting Sri Lankan mud crabs was because of the taste.

Table 8 shows alternative options to mud crabs. The majority of the respondents would like to have other crabs (king or blue crabs, etc.) or shrimp/prawn.

## 5. Discussion

Three main findings were obtained from this paper. First, the conditional logit model results indicated that respondents preferred to eat mud crabs from Sri Lanka. This result is consistent with the market information available at various online store sites in Singapore that recognized them as ‘Sri Lankan crab’. Previous studies by Loose et al. in 2013 discussed that the country of origin is a common attribute in food purchasing decisions, and, Sharma et al. in 1995 also concluded that regionalism and ethnocentrism are vital factors for regional preferences [18]. A study by Scarpa et al. in 2005 suggested that country of origin plays a vital role as an indicator in terms of quality for taste features in the specific region [17]. This study reinforces previous findings that the country of origin of food matters.

Second, the analysis of Fisher’s exact test shows that religion is associated with the consumption of male or female mud crabs. This study is one of the first that evaluates different religions and their different food preferences. The post hoc analysis (adjusted residual analysis in Table 6) shows that respondents who identify as Christian significantly preferred to eat female mud crab without eggs and participants from the Hindu religion tended to prefer male mud crabs. Cohen [27] mentioned that different religions have different rules and these have changed over time. In this study, it was found that different religions have different preferences for male and female mud crabs. However, this research had limitations in knowing whether eating crabs under specific conditions is permitted in any religion or not. Further research is required to understand the reasons why specific religions influence the potential consumption of specific food items.

Third, this study adds a new perspective for regional differences in food preferences. Previously, Zhang discussed that mud crab consumers in Maryland, USA preferred male crabs because they contain more meat. She also added that female crabs cost less because of the size and demand, and stated that consumers did not like the roe inside the female crabs [10]. In this study, the parameter estimation in the conditional logit model of female mud crabs with eggs, and female mud crabs without eggs provides no statistically significant results. This is because 55% of the respondents answered that they prefer all types of (including male and female) mud crabs (Table 5) and, therefore, no significant distinction of the preference between male and female crabs can be identified. At the same time, it should be noted that more respondents in Singapore answered that they prefer female mud crabs (including with and without eggs) over male mud crabs (Table 5). These findings suggest that consumers in Singapore could show a preference for female crabs, if compared with those in Maryland, USA, who showed a preference for male mud crabs. Further studies should be conducted to compare the consumers in the USA with Asian countries to discover if culture or food habits are the main issues while consuming different sex of crabs.

## 6. Limitations of the Study

This paper does not estimate the other models, such as the multinomial model, mixed model, and nested logit models. It is well-known that the conditional logit model requires the Independence of Irrelevant Alternatives (IIA) condition. A potential concern is that IIA indicates that unobservable components of utility within nine choice experiments by the same participants are independent. However, if a respondent is in a bad mood in the first-choice experiment, he/she is likely in the same mood until the last choice experiment, which results in a correlation between *e* for the same person over choice experiments. To deal with these concerns, the authors should have estimated three other models that do not require IIA. Moreover, the research was conducted in only one Southeast Asian country, which was Singapore, due to the shortage of time and also due to pandemic situations. Furthermore, ethnicity was not the focus of this study and not including ethnicity did not invalidate the results of this study.

## 7. Conclusions

It was found in this research, that country of origin affects consumer’s purchasing decisions. Respondents showed preferences for mud crabs from Sri Lanka. ‘Taste’ is the foremost important factor for consuming mud crabs. Moreover, consumers are willing to pay more for live mud crabs and only pay half for frozen mud crabs, compared to fresh, but not alive, mud crabs. Furthermore, this study identified that religion is associated with the consumption of either male or female mud crabs. Christian consumers tend to prefer female mud crabs without eggs. Also, Hindu consumers prefer to consume male mud crabs. Currently, the mud crab industry is growing in Asia, and the rate of consumption is also increasing in this region. Sustainability of mud crab productions could be better maintained if the exact demand of different types of crabs (such as male, female without eggs, or female with eggs) are known, because overharvesting of female crabs with eggs could cause more damage to the crab population. This study obtained useful information regarding the sustainable consumption of mud crabs in the region through the accurate evaluations of consumer preferences.

## Figures and Tables

**Table 1:** Attributes and levels in the DCE.

Attribute	Level
Country of origin	Cambodia, Indonesia, Sri Lanka
Sex of the mud crab	Male, Female with eggs, Female without eggs
Condition of the mud crab	Alive mud crabs, Fresh but not alive mud crabs, Frozen (whole) mud crabs
Price	50 SGD, 60 SGD, 70 SGD

**Table 2:** An example of choice sets from the Discrete Choice Experiment (DCE).

Attributes	Option 1	Option 2	Option 3	Option 4
Country of origin	Cambodia	Cambodia	Indonesia	Neither
Sex of the crab	Female mud crab with eggs	Female mud crab with eggs	Female mud crab with eggs	
Condition of the crab	Frozen mud crab (whole)	Fresh but not alive mud crab	Frozen mud crab (whole)	
Price	50 SGD	50 SGD	60 SGD	

Example of choice set for choice experiment. Notes: Each subject is asked to select one of the 3 alternatives with 4 attributes and an opt-out alternative. There are 9 questions in total with 3 alternatives in each question and an opt-out alternative.

**Table 3:** Demographics of participants (*n* = 312).

Variable	Frequency	In Percentage
**Gender**		
Female	123	39.4
Male	189	60.6
**Age**		
18–25	75	24.0
26–35	124	39.7
36–45	82	26.3
46–55	14	4.5
55 & above	17	5.4
**Religion**		
Atheism	1	0.3
Buddhism	77	24.7
Christianity	77	24.7
Hinduism	38	12.2
I decline to answer	12	3.8
Islam	46	14.7
Judaism	1	0.3
No religion	43	13.8
Sikhism	4	1.3
Taoism	13	4.2
**Profession**		
Employed	194	62.2
Housewife	1	0.3
Retired	1	0.3
Self-Employed	39	12.5
Student	59	18.9
Unemployed	18	5.8
**Family members**		
2 to 3	137	43.9
4 to 5	129	41.3
Alone	8	2.6
More than 5	38	12.2
**Income**		
Confidential	63	20.2
Less than S$ 5000	65	20.8
More than S$ 20,000	29	9.3
No Income	11	3.5
Not Sure	25	8.0
S$10,000 to S$20,000	37	11.9
S$5000 to S$ 10,000	82	26.3

**Table 4:** Results from conditional logit model and willingness to pay estimate (SGD, Singaporean dollars).

	Estimate	Robust Standard Error	*p* Value (2 Sided)	Willingness to Pay Estimate
ASC	0.88	0.24	1.93 × 10^−4^ ***	-
Indonesia	−0.31	0.06	8.74 × 10^−7^ ***	−16.48
Cambodia	−0.32	0.06	4.93 × 10^−7^ ***	−16.97
Female mud crab with eggs	−0.03	0.06	0.62	−1.68
Female mud crab without eggs	−0.09	0.06	0.13	−4.62
Alive mud crabs	0.47	0.09	5.19 × 10^−8^ ***	25.08
Frozen mud crabs	−0.25	0.08	1.06 × 10^−3^ ***	−13.40
Price of the mud crab	−0.02	0.003	2.17 × 10^−8^ ***	-
Number of observations (sample size)	2808 (*n* = 312)	-	-	-
Log likelihood of model	3755.23	-	-	-
McFadden’s pseudo R^2^	0.04	-	-	-
AIC	7526.47	-	-	-
BIC	7574.01	-	-	-

**Note:** *** *p* < 0.01.

**Table 5:** Marginal cross table between religion and consumption of male or female mud crabs.

TOTAL	171	53	13	20	39	16	312
I decline to answer	10	1	1	0	0	0	12
Judaism	0	0	0	0	1	0	1
Sikhism	2	0	2	0	0	0	4
Taoism	9	2	0	1	1	0	13
No religion	21	11	0	3	5	3	43
Hinduism	22	3	0	6	5	2	38
Buddhism	43	18	0	1	10	5	77
Christianity	42	13	7	6	7	2	77
Islam	22	5	3	3	9	4	46
Atheism	0	0	0	0	1	0	1
	All of them	Female mud crab with eggs	Female mud crab without eggs	Male mud crab	Never ate mud crabs	None of them	TOTAL

**Table 6:** Adjusted residual analysis between the religion and preference of consumption of mud crabs.

Atheism	−0.74	−0.41	−0.20	−0.25	2.48	−0.23
Buddhism	0.12	1.36	−1.79	−1.77	0.12	0.53
Christianity	−0.03	−0.02	2.12	0.48	−0.85	−0.98
Hinduism	0.26	−1.36	−1.26	2.28	0.12	0.04
I decline to answer	1.34	−0.73	0.71	−0.88	−1.23	−0.78
Islam	−0.64	−1.01	0.78	0.03	1.36	1.07
Judaism	−0.74	−0.41	−0.20	−0.25	2.48	−0.23
No religion	−0.53	1.37	−1.34	0.15	−0.16	0.54
Sikhism	−0.13	−0.82	4.49	−0.51	−0.71	−0.45
Taoism	0.70	−0.14	−0.74	0.18	−0.49	−0.82
	All of them	Female mud crab with eggs	Female mud crab without eggs	Male mud crab	Never ate mud crabs	None of them

**Table 7:** The preference of the country of origin of mud crabs that participants chose to eat.

Country	Number of Respondents
Australia	20
Bangladesh	42
Cambodia	63
China	33
India	32
Indonesia	58
Malaysia	99
Myanmar	34
Philippines	42
Singapore	112
Sri Lanka	115
Taiwan	18
Others	0

**Table 8:** Alternative to mud crabs.

Alternative Options to Mud Crabs	Number of Respondents
Shrimp/Prawn	86
Other crabs (King crabs, blue crabs, etc.)	88
Chicken	55
Fish	38
Can’t be replaced	36
Others	5

## Data Availability

All data provided by Aperion (the survey company in Singapore) to the University of Tokyo is kept by the Laboratory of Global Fishery Sciences, Graduate School of Agricultural and Life Sciences, The University of Tokyo.
